# Post-COVID-19 irritable bowel syndrome: an integrative review

**DOI:** 10.1590/0100-6991e-20233618-en

**Published:** 2023-11-10

**Authors:** Julyanne Tereza Cordeiro Silva, Olival Cirilo Lucena da Fonseca

**Affiliations:** 1 - Centro Universitário Maurício de Nassau, Recife - PE - Brasil; 2 - Hospital Universitário Oswaldo Cruz, Serviço de Cirurgia Geral e Transplante de Fígado - Recife - PE - Brasil

**Keywords:** Irritable Bowel Syndrome, COVID-19, SARS-CoV-2, Síndrome do Intestino Irritável, COVID-19, SARS-CoV-2

## Abstract

**Introduction:**

the persistence of long-term symptoms of COVID-19 represents a new challenge for the medical-scientific community, it is the condition called long-term COVID-19. Irritable Bowel Syndrome (IBS) is one of the most common Disorders of the Gut-Brain Interaction and its post-infection development is already validated. According to the Rome IV criteria, it is characterized by the presence of recurrent abdominal pain, on average, at least 1 day a week in the last 3 months with onset of symptoms at least 6 months before diagnosis, associated with 2 or more factors: related to defecation and/or associated with change in stool frequency and/or associated with change in stool form. This study aimed to review data on post-COVID-19 IBS.

**Methods:**

this is an integrative review of studies published between January 1, 2020 and April 30, 2023, which presented data on IBS with previously diagnosed COVID-19 disease. The PubMed database was used, the descriptors were “Irritable bowel syndrome” AND “COVID-19”; the reference list of the articles was also retrieved.

**Results:**

eight studies were reviewed, it was observed that 0.6% to 11.6% of patients had IBS again after a minimum period of 6 months of infection. Risk factors were female gender, severity of COVID-19, presence of acute-phase gastrointestinal symptoms, and depression/anxiety.

**Conclusion:**

the results obtained suggest that COVID-19 may be associated with the emergence of de novo IBS. Further studies are needed to investigate its long-term effects and clinical spectra.

## RESUMO

**Introdução:**

a persistência de sintomas a longo prazo da COVID-19 representa um novo desafio à comunidade médico-científica, é a condição denominada COVID-19 longo. A Síndrome do Intestino Irritável (SII) é um dos Distúrbios da Interação Intestino Cérebro mais comuns e seu desenvolvimento pós-infecção já é validado. Segundo os critérios de Roma IV, caracteriza-se por presença de dor abdominal recorrente, em média, pelo menos 1 dia por semana nos últimos 3 meses com início dos sintomas pelo menos 6 meses antes do diagnóstico, associada a 2 ou mais fatores: relacionada à defecação e/ou associada com mudança na frequência das fezes e/ou associada com mudança na forma das fezes. Este estudo teve como objetivo revisar dados acerca de SII pós-COVID-19.

**Métodos:**

trata-se de uma Revisão Integrativa de estudos publicados entre 1º de janeiro de 2020 a 30 de abril de 2023, que apresentassem dados acerca de SII com a doença COVID-19 previamente diagnosticada. Utilizou-se a base de dados PubMed, os descritores foram “Irritable bowel syndrome” AND “COVID-19”; a lista de referência dos artigos também foi recuperada.

**Resultados:**

oito estudos foram revisados, observou-se que 0,6% a 11,6% pacientes cursaram com SII de novo após um período mínimo de 6 meses da infecção. Os fatores de risco foram sexo feminino, gravidade da COVID-19, presença de sintomas gastrointestinais de fase aguda e depressão/ansiedade.

**Conclusão:**

os resultados obtidos permitem crer que a COVID-19 pode estar associada ao surgimento de SII de novo. São necessários outros estudos para investigar seus efeitos a longo prazo e espectros clínicos..

## INTRODUCTION

After the initial outbreak in the city of Wuhan in China at the end of 2019^1^, COVID-19 crossed borders and became a major challenge for global health authorities and teams. Initially, it was believed that the SARS-CoV-2 virus was limited only to the respiratory tract^2^, but it did not take long for the first descriptions of extrapulmonary manifestations. Around half of patients at the epicenter of the pandemic already reported one or more digestive symptoms, such as anorexia, diarrhea, vomiting, and abdominal pain during the infection acute phase^3^.

In addition to the high morbidity and mortality worldwide, a new clinical spectrum of this disease has been debated by the medical-scientific community^4^. It has been noticed that a portion of surviving patients may suffer from long-term sequelae, be they pulmonary, hematological, cardiovascular, endocrine, dermatological, neuropsychiatric, renal, or gastrointestinal^4^ ^,^ ^5^. It is the condition currently defined as “long COVID-19”, characterized by the persistence or development of symptoms after acute infection that cannot be explained by an alternative diagnosis^4^ ^-^ ^6^. Any patient can develop long COVID-19 and the symptoms vary in severity, temporality, and affected systems^4^ ^,^ ^6^.

Interestingly, it has been observed that patients previously diagnosed with COVID-19 are at greater risk of developing Irritable Bowel Syndrome (IBS) when compared with uninfected individuals^7^. IBS, in turn, is one of the Gut-Brain Interaction Disorders (GBID), previously called Functional Gastrointestinal Disorders (FGD)^8^. Currently, the diagnosis is based on the Rome IV criteria^8^. They are related to any combination of motility disorders, visceral hypersensitivity, mucosal and immune dysfunction, altered intestinal microbiota, and altered central nervous system processing, and result in significant global healthcare costs and substantial impairments to quality of life^9^.

Post-infection IBS (PI-IBS) is already recognized in the literature and can develop in around 10% of patients after infectious enteritis^10^. The pathophysiological mechanisms that would lead to COVID-19 subsequently triggering IBS are still poorly understood. Enterocytes express angiotensin-converting enzyme (ACE-2) receptors, a receptor used by the virus to enter human cells^11^, which would explain its intestinal tropism. Other justifications include increased intestinal permeability, chronic hyperinflammatory state, and changes in the intestinal microbiota^12^ ^,^ ^13^, consistent with what is already known about PI IBS^9^.

Thanks to the large body of evidence about COVID-19 and the emergence of vaccines, the mortality rate has been reduced worldwide. However, the long-term effects of COVID-19 are a current healthcare challenge and are still poorly understood.

## GOAL

Give this scenario and the impact of IBS on an individual’s quality of life, this study aims to review data on post-COVID-19 IBS.

## METHODS

This study is configured as an Integrative Review, outlined according to the steps: identification of the problem with elaboration of a guiding question; literature research; evaluation and analysis of data; and presentation of the review with its results and limitations^14^. Development of Irritable Bowel Syndrome post-COVID-19: is it possible?

The primary search was carried out in the PubMed database, with the terms “Irritable bowel syndrome” AND “COVID-19”, available on MeSH and DeCS. The reference list of the articles found was also manually retrieved, looking for additional relevant studies.

### Eligibility Criteria

We selected prospective or retrospective, single-arm or comparative, single-center or multi-center studies, published between January 1, 2020, and April 30, 2023, in English, which presented data on Irritable Bowel Syndrome with COVID-19 disease previously diagnosed in adult patients, the diagnosis of IBS necessarily using the Rome IV criteria and laboratory-confirmed COVID-19, regardless of the presence of symptoms.

The diagnosis of IBS according to the Rome IV criteria requires presence of recurrent abdominal pain, on average, at least one day per week in the last three months, with onset of symptoms at least six months before diagnosis, associated with two or more of the following: related to defecation and/or associated with changes in stool frequency and/or associated with changes in the form of stools^8^.

We also required a minimum period of six months of follow-up of the entire sample studied and an interval of at least six months between the diagnosis of COVID-19 and the interview with patients for inclusion.

We excluded case reports and series, letters, pre-publications, reviews, studies without full text available, and studies with patients already diagnosed with IBS prior to SARS-CoV-2 infection, which reported only worsening of symptoms and not a diagnosis of new IBS, or that used previous Rome Criteria (III or II). We also discarded studies approaching IBS concomitantly with the COVID-19 pandemic, without necessarily associating it with the viral infection.

**Figure 1:**
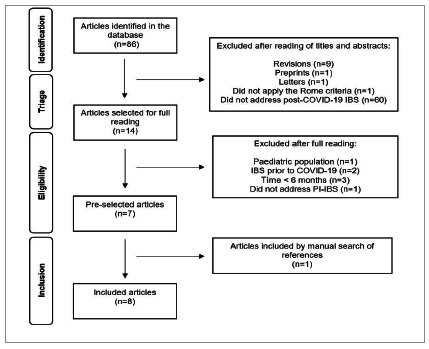
Flowchart of the study selection process.

Initially, we identified 86 studies. After reading the titles and abstracts, we excluded 72 of them. Of the 14 chosen for full reading, we excluded seven and retrieved one from the list of references. The final sample for the review consisted of eight studies. (Figure 1).

## RESULTS

The eight included studies were organized according to publication order, from oldest to most recent, dating from March 2022 to March 2023. We extracted general data related to the design (Table 1) and main results. All authors reported that patients positive for COVID-19 were laboratory tested.

**Table 1:** General characteristics of the included studies.

Study / 1^st^ Author	Month, year	Study design	Time (months)	Number of cases/controls	Frequency of IBS cases/controls
E1 / Ghoshal et al.^15^	Mar, 2022	Prospective, case-control	6	280 / 264	20 (7,1 %) / 1 (0,38%)
E2 / Vélez et al.^16^	Jun, 2022	Prospective, longitudinal cohort	6	200 / -	21 (10,5%) / -
E3 / Austhof et al.^17^	Jul, 2022	Prospective, longitudinal cohort	6	1475 (only 49 completed the Rome IV survey) / -	5 (10,2) / -
E4 / Nazarewska et al.^12^	Nov, 2022	Prospective, longitudinal	6	257 / -	15 (5,8%) / -
E5 / Farsi et al.^18^	Dec, 2022	Prospective, transversal	6	233 / -	27 (11,6%) / -
E6 / Marasco et al.^19^	Dec, 2022	Prospective, case-control	6 12	548 / 224 435 / 188	3 (0,6%) / 2 (0,9%) 14 (3,2%) / 1 (0,3 %)
E7 / Siyal et al.^20^	Jan, 2023	Prospective, longitudinal	6	303 / -	32 (10,6%) / -
E8 / Golla et al.^21^	Mar, 2023	Prospective, cohort	6	320 / 320 (group A) and 280 (group B)	NR / 0 group A and B

E: study; Cases: patients with a previous diagnosis of COVID-19; Controls: patients without a diagnosis of COVID-19; Time: follow-up or interval between COVID diagnosis and research; NR: not reported.

The first study (E1)^15^ aimed to prospectively evaluate the frequency and spectrum of FGIDs among 280 post-COVID-19 patients and 264 healthy controls and the risk factors involved. They used the Rome III criteria at first and later compared the results to Rome IV, which is why it was included. Patients with a previous history of FGID, history of abdominal surgery, severe psychiatric illness, inflammatory bowel disease (IBD), gastrointestinal cancer, and critical COVID-19 were excluded. COVID-19 patients were comparable to healthy controls as to age and sex. They were followed up physically or by telephone at one, three, and six months.

At six months of follow-up, 15 patients with COVID-19 (5.3%) developed IBS, six (2.1%) UD (uninvestigated dyspepsia), and five (1.8%), overlapping IBS-UD. Only one healthy control developed IBS within six months (p<0.05 for all except IBS-UD overlap). Of these 15 patients with IBS only diagnosed by Rome III criteria, 14 (93%) met Rome IV criteria. Of all 20 patients with IBS (including five with overlap), 12 (60%) were diarrhea-predominant, four (20%) were constipation-predominant, and the other four (20%) had unclassified IBS. Of the 280 patients with COVID-19, 164 (58.6%) were symptomatic and 116 (41.4%) asymptomatic. At six months, the symptomatic patients developed FGID more frequently (IBS 13/164 [7.8%] vs. 2/116 [1.7%], p=0.03; UD 6/164 [3.7%] vs. 0/116 [0%], p=0.05; and IBS-UD overlap 5/164 [3%] vs. 0/116 [0%], p=0.07). Furthermore, the presence of gastrointestinal symptoms during COVID-19 baseline was more frequently associated with the development of FGID also during the 6-month follow-up^15^.

The second study (E2)^16^ included outpatients diagnosed with COVID-19 who underwent a standard, institution-specific screening of gastrointestinal symptoms during April to September 2020. These patients were evaluated by telephone six months after COVID-19 diagnosis, using the Rome IV criteria in search of functional dyspepsia (FD) and PI IBS. Patients who reported similar symptoms before infection or organic conditions that explained gastrointestinal symptoms were excluded. Among the 200 patients in the sample, 79 (39.5%) developed new dyspeptic and chronic intestinal disorders post-COVID-19, 58 of these with FD, two with IBS only, and 19 with associated FD and IBS. After multivariate adjustment, female sex and a history of depression or anxiety were seen as independent risk factors for the incidence of FD and IBS, whereas the presence of gastrointestinal symptoms at the diagnosis of COVID-19 was not related to post-COVID-19 gastrointestinal complaints^16^.

E3^17^ examined the association between the presence of gastrointestinal symptoms during SARS-CoV-2 infection and the persistence of gastrointestinal symptoms (≥45 days), as well as the development of PI-IBS. Austhof et al.^17^ defined two groups of participants, one that presented gastrointestinal symptoms during the acute infection and another that did not. Gastrointestinal symptoms were defined as diarrhea, nausea, or vomiting. Data were acquired through self-reported online surveys. Those who reported gastrointestinal symptoms during the acute phase were asked to complete the Rome IV diagnostic questionnaire for IBS at six weeks and three and six months after the first survey. Participants who reported previous gastrointestinal conditions such as IBS, IBD, Crohn’s disease or ulcerative colitis, chronic diarrhea, chronic constipation, dyspepsia, or reflux disease were excluded. Of the 1,475 patients in the sample, 33.8% (n=499) presented gastrointestinal symptoms during the acute infection. Cases with acute gastrointestinal symptoms had four times greater odds of persistent gastrointestinal symptoms than cases without acute gastrointestinal symptoms (OR 4.29, 95% CI 2.45 7.53), even after adjustment for age, sex, and stress. Only 49 patients completed the Rome IV scale, five of these (10.2%) met the diagnosis for PI-IBS. Overall, PI-IBS was reported on average 6.2 months after acute infection^17^.

The fourth study (E4)^12^ was a prospective evaluation of patients previously hospitalized for COVID-19 to determine the prevalence of PI-IBS and its risk factors. The Rome IV criteria were applied immediately after hospital discharge and after three and six months of follow-up. A total of 257 patients who required hospitalization were included. Among them, 146 (56.8%) reported gastrointestinal symptoms during the COVID-19 acute phase. Gastrointestinal symptoms (abdominal pain with diarrhea or constipation) that did not meet the criteria for duration of Symptoms for IBS were reported in 28 (10.6%), 58 (22.3%), and 70 (26.9%) patients at hospital discharge, and after three and six months of follow-up, respectively. At six months, 15 participants (5.8%) met the criteria for IBS; none had a prior history of COVID-19. The authors also applied the modified Rome IV criteria^22^ and the IBS rate after six months increased to 26.9%. They found no correlation between gastrointestinal symptoms during COVID-19, comorbidities, and treatment used during hospitalization.

E5^18^ investigated the incidence of IBS and its correlation with the state of stress in patients diagnosed with COVID-19 between November 2020 and February 2021. Patients with asymptomatic COVID-19, those admitted to the ICU in critical condition, with neurological diseases, and gastrointestinal disorders were not included in the analysis. The sample consisted of 233 patients. After a minimum period of six months after infection, they were subjected to stress and Rome IV questionnaires. The rate of IBS was 11.6% and its proportion was higher in women and in people aged 40 and over. Symptoms of depression and anxiety were described by 27.4% and 36.9% of patients, respectively. There was no significant difference between participants in terms of COVID-19 severity, BMI, smoking status, and medical history. The odds of depression and anxiety were higher in COVID-19 patients with IBS symptoms, but not statistically significant.

E6^19^ is a multicenter, controlled study that evaluated the presence of gastrointestinal symptoms, anxiety, and depression in patients with and without COVID-19 at hospital admission and at one, six, and 12 months after admission. The primary outcome was the incidence of post-COVID-19 IBS, according to the Rome IV criteria. The study was conducted at 36 centers in 14 countries. Recruitment took place from May to October 2020. Data from 883 patients (614 COVID-19 and 269 controls) were used for baseline assessments. Follow-up assessments were completed by 772 patients (548 COVID-19 and 224 controls) in six months and by 623 patients (435 COVID-19 and 188 controls) in 12 months. At six months, only three COVID-19-positive patients met the IBS criteria, there being no statistical significance (p= 0.587). At 12-month follow-up, patients with COVID-19 had significantly higher IBS rates compared with controls (3.2% vs 0.5%, p=0.045). Among the 14 patients with COVID-19 who developed IBS, four (28.6%) reported IBS-C (constipation-predominant IBS), seven (50%) IBS-D (diarrhea-predominant IBS), one (7.1%) IBS-M (IBS with mixed habits), and two (14.3%) IBS-U (IBS not classified). Previous diagnosis of COVID-19 was a predictive factor for the occurrence of IBS in the entire cohort (OR = 10.686). The risk of IBS among COVID-19 cases was higher among patients with a history of allergies, chronic intake of proton pump inhibitors, and dyspnea upon hospitalization. Patients with COVID-19 also showed higher levels of depression and anxiety at six and 12 months after hospitalization^19^.

The seventh study (E7)^20^ evaluated the incidence of IBS in post-COVID-19 patients and its predisposing factors. Those with a history of IBS prior to infection, IBD, colitis, celiac disease, gastrointestinal neoplasia, severe psychiatric illness, and any other condition that could mimic IBS symptoms were excluded. A total of 303 patients were included in the study and were followed prospectively. Those who presented symptoms similar to IBS were asked to undergo follow-up at gastrointestinal clinics at one, three, and six months, where laboratory investigations were carried out when relevant and those aged 45 and over underwent colonoscopy. At six-month follow-up, 32 (10.6%) patients met Rome IV criteria for IBS. Among them, 17 (53.13%) had IBS-D, 10 (31.25%) had IBS-C, and five (15.62%) had IBS-M. Twenty-four (75%) patients were female. Independent risk factors for post-COVID-19 IBS found were need for oxygen during hospitalization (p=0.016), concomitant gastrointestinal symptoms during infection (p<0.001), female sex (p<0.001), and high levels of procalcitonin (p=0.017) during hospitalization.

The eighth and final included study (E8)^21^ prospectively studied the frequency, spectrum, and risk factors for FGIDs after COVID-19 diagnosis compared with uninfected controls. The study was conducted from April 2021 to January 2022. Monitoring was carried out in person or by telephone for one, three, and six months, using a questionnaire according to the Rome IV criteria. Cases and controls with IBD, severe psychiatric illness, gastrointestinal neoplasia, history of abdominal surgery, or on immunosuppressive therapy were excluded. They included 320 cases with COVID-19 and two control groups: group A = 320 healthy spouses/family members sharing the same dietary and environmental factors, and group B = 280 healthy controls with negative serology for COVID-19. The authors reported that at three months, eight (2.5%) patients had symptoms consistent with IBS and two (0.6%), IBS FD, as well as other FGID. The actual rate of IBS at six months was not specified, but FGIDs overall were present in about 7% of the COVID-19 patients analyzed. None of the healthy controls developed FGID within six months of follow-up (p<0.01). Moderate-severe COVID-19 and the presence of gastrointestinal symptoms during the acute phase of infection and at one month were predictors for the development of FGID (p<0.01).

## DISCUSSION

Post-COVID-19 symptoms are complex and heterogeneous and there is still no consensus on the definition of long COVID. César Fernández de las Peñas proposed a definition that considers the timeline in which symptoms appear and/or disappear and the nature of the symptoms: “Long COVID should be used to define the presence, in general, of any post-COVID symptoms after overcoming the SARS-CoV-2 infection and will consist of two stages: 1) post-acute sequelae of SARS-CoV-2 infection (PASC) or acute post-COVID - from week five to week 12 after the onset of symptoms; and 2) chronic post-COVID - duration longer than 12 weeks.” He also points out the need to determine the pattern (fluctuating or persistent) and the nature of each specific symptom^23^. The National Institute for Health and Clinical Excellence (NICE) defines long COVID as symptoms that continue or develop after acute SARS-CoV-2 infection and that cannot be explained by an alternative diagnosis; it includes 1) ongoing symptomatic COVID-19 - from four to 12 weeks after infection; and 2) post COVID 19 syndrome - beyond 12 weeks^4^. The definition from the US Centers for Disease Control and Prevention describes long COVID as sequelae that extend beyond four weeks after the initial infection^4^.

PI-IBS is not new in science, its first formal description having been published in 1962 by Chaudhary and Truelove^24^. A recent systematic review of 45 studies, including 21,421 individuals with enteritis followed for three months to 10 years, found a pooled prevalence of IBS of 10.1% at 12 months after infectious enteritis^25^.

The post-COVID-19 IBS rate among the eight included studies ranged from 0.6%^19^ to 11.6%^18^. Some studies criticized the Rome IV criteria due to their low sensitivity. In 2022, the Rome Foundation proposed a modification of the criteria for clinical practice, where a diagnosis of FGID can still be made if 1) the nature of the symptoms matches the Rome IV FGID diagnostic criteria and 2) the symptoms interfere with quality of life. Therefore, a lower frequency and shorter duration (eight weeks or more) than those required by traditional criteria are allowed if there is clinical confidence that other diagnoses have been sufficiently ruled out^22^. In E4^12^, the post-COVID-19 IBS rate increased from 5.8% to 26.9% at six months. However, for research purposes the standard Rome IV criteria need to be followed^22^.

In general, female sex, clinically severe enteritis, use of antibiotics during treatment, and psychological distress are associated with a higher risk of PI-IBS^25^. The results regarding the risk factors for long COVID-19 are quite conflicting, some authors already arguing that any patient can develop it, regardless of infection severity and received treatment^4^.

In three studies (E1^15^, E5^18^, and E7^20^), female sex was more associated with the development of post-COVID-19 IBS.

Regarding the severity of COVID-19, while in E8^21^ moderate-severe COVID-19 was associated with the development of FGID (p<0.01), E5^18^ did not identify a significant difference between participants in terms of severity of COVID-19, but there was no comparison with controls. COVID-19 severity has not yet been validated as an independent predictor for PI-IBS, but Yamamoto et al. observed that more patients with IBS presented severe COVID-19 (requiring ICU admission) than those without IBS^26^. Differences in the samples studied make a reliable general analysis impossible. E2^16^, for example, included only outpatients, with the justification that they could have a clearer assessment of gastrointestinal symptoms during the infection.

Another important factor that has been researched is whether the presence of gastrointestinal symptoms during the acute phase of SARS-CoV-2 infection might predispose the development of gastrointestinal disorders in the long term. In E3, Austhof and colleagues noticed that cases with acute gastrointestinal symptoms during COVID-19 had a four times greater chance of persistent gastrointestinal symptoms than those without acute gastrointestinal symptoms^17^. Three works (E1^15^, E7^20^, and E8^21^) highlighted the presence of gastrointestinal symptoms in the acute phase as predictors for IBS, whereas in studies E2^16^ and E4^12^ there was no correlation. It is worth noting that most of the studies included were based on self-reports, requiring patients to be able to recall their acute phase symptoms.

Psychological factors were also evaluated by some and should be considered due to the impact brought by the COVID-19 pandemic on lifestyle, especially in the first waves^13^. Even with a specific viral activity confirmed, they may have even contributed to aggravating factors. History of depression or anxiety were seen as independent risk factors for the incidence of IBS in E2^16^, being also present in 27.4% and 36.9% of patients in E5^18^, respectively, and patients with COVID-19 in E6^19^ also showed higher levels of depression and anxiety at six and 12 months after hospitalization.

It is believed that there are no unique pathophysiological mechanisms for PI-IBS itself, but rather an interaction between central and peripheral factors, including changes in the intestinal microbiota, epithelial and immune dysfunction, mucosal inflammation, and neuromotor mechanisms^10^.

To enter human cells, SARS-CoV-2 uses the ACE-2 receptor, widely expressed in cells of the gastrointestinal tract^11^. The infection triggers direct cytotoxic damage, inducing apoptosis and increased intestinal permeability^13^ ^,^ ^19^. Increased permeability is considered an early event, associated with low-grade immune activation, which may be present in patients with PI-IBS^10^.

It is also postulated that the hyperinflammatory state of the COVID-19 acute phase may persist and contribute to the emergence of symptoms in the long term, especially PI-IBS^13^ ^,^ ^19^. The innate immune system has been described as altered in the intestinal mucosa of patients with PI-IBS^10^. Recently, it was noted that patients with long COVID-19 may present highly active innate immune cells and an elevated expression of type I and III interferons, even eight months after infection^27^.

Another triggering factor advocated is the change in the intestinal microbiota post COVID-19^13^, with an increase in opportunistic bacteria and a decrease in useful commensals^12^. Individuals who developed IBS may have been unable to overcome post-infectious dysbiosis^10^. Finally, psychological components such as stress, anxiety, and depression are also considered predisposing to post-COVID-19 IBS^13^. 

### Applicability

The fact that long COVID-19 is a condition that is still not widespread and is quite recent makes this study even more relevant, as it provides additional evidence about the problem. A positive point is that the included studies were based on the Rome IV criteria and even the one that initially used the III subsequently compared the results^15^. It is also worth noting that the inclusion of only original studies ends up providing greater insights into the incidence and associated risk factors.

Despite a small sample size when compared with other studies, our criteria were quite strict in looking for the presence of new-onset IBS. During the search, many manuscripts included patients previously diagnosed with IBS and classified COVID-19 only as a factor in worsening symptoms, some of which were even included in certain reviews. Others reported an increase in the IBS rate during the pandemic, without necessarily being associated with a positive laboratory diagnosis, which could, erroneously, underestimate or overestimate the real PI-IBS rate.

### Limitations

Our review is not without limitations. We did not review the studies systematically and multivariate analyzes were not performed. Furthermore, as it is a new challenge for health systems and the scientific community, the studies are quite heterogeneous in terms of methodology and, consequently, this affects results. Not all risk factors were evaluated equally across studies and few included control groups. The presence of depression and anxiety, known predisposing factors for IBS, may also have been the cause of the development of the syndrome in these patients and cannot be ruled out. In most of them, the interviews were carried out by telephone and online questionnaires, leaving room for memory bias and increased subjectivity.

## CONCLUSION

The results obtained from this review suggest that COVID-19 may be associated with the emergence of new IBS. However, larger, prospective, and controlled studies are needed to investigate the long-term effects of COVID-19, its underlying pathophysiology, and its appropriate management.
